# HGF–MET Cascade, a Key Target for Inhibiting Cancer Metastasis: The Impact of NK4 Discovery on Cancer Biology and Therapeutics

**DOI:** 10.3390/ijms14010888

**Published:** 2013-01-07

**Authors:** Shinya Mizuno, Toshikazu Nakamura

**Affiliations:** 1 Division of Virology, Department of Microbiology and Immunology, Osaka University Graduate School of Medicine, 2-2-B7 Yamadaoka, Suita 565-0871, Japan; E-Mail: mizuno@onbich.med.osaka-u.ac.jp; 2 Division for Regenerative Drug Discovery, Center for Advanced Science and Innovation, Osaka University, 2-1 Yamadaoka, Suita 565-0871, Japan

**Keywords:** HGF, MET, NK4, oncogenesis, cancer invasion and metastasis, angiogenesis, perlecan

## Abstract

Hepatocyte growth factor (HGF) was discovered in 1984 as a mitogen of rat hepatocytes in a primary culture system. In the mid-1980s, MET was identified as an oncogenic mutant protein that induces malignant phenotypes in a human cell line. In the early 1990s, wild-type MET was shown to be a functional receptor of HGF. Indeed, HGF exerts multiple functions, such as proliferation, morphogenesis and anti-apoptosis, in various cells via MET tyrosine kinase phosphorylation. During the past 20 years, we have accumulated evidence that HGF is an essential conductor for embryogenesis and tissue regeneration in various types of organs. Furthermore, we found in the mid-1990s that stroma-derived HGF is a major contributor to cancer invasion at least *in vitro*. Based on this background, we prepared NK4 as an antagonist of HGF: NK4 inhibits HGF-mediated MET tyrosine phosphorylation by competing with HGF for binding to MET. *In vivo*, NK4 treatments produced the anti-tumor outcomes in mice bearing distinct types of malignant cancers, associated with the loss in MET activation. There are now numerous reports showing that HGF-antagonists and MET-inhibitors are logical for inhibiting tumor growth and metastasis. Additionally, NK4 exerts anti-angiogenic effects, partly through perlecan-dependent cascades. This paper focuses on the chronology and significance of HGF-antagonisms in anti-tumor researches, with an interest in NK4 discovery. Tumor HGF–MET axis is now critical for drug resistance and cancer stem cell maintenance. Thus, oncologists cannot ignore this cascade for the future success of anti-metastatic therapy.

## 1. Introduction

Cancer has become increasingly common among the aged. A report of the worldwide incidence and mortality from 27 kinds of tumors in 2008 estimates 12.7 million new cases and 7.6 million deaths occurred in 2008 [1]. The difference between benign and malignant tumors depends on the absence or presence of metastatic features. In benign tumors, neoplastic growth is limited within the tissue of origin, particularly within the basement membrane, composed of extra-cellular matrix (ECM), which acts as a biological barrier. In contrast, malignant tumors acquire some properties involved in cancer cell dissociation, invasion and metastasis, and this response is mediated via the destruction of ECM-based barriers by matrix metalloproteinase (MMP). The invasive tumor cells successfully metastasize to distant organs via blood flow. Thus, it is important to elucidate the molecular basis of cancer metastasis.

Hepatocyte growth factor (HGF) was discovered as a potent mitogen of rat hepatocytes in primary culture [2–4]. Beyond its name, HGF is now recognized as an essential organotrophic factor in almost all tissues [4–8]. HGF induces mitogenic, motogenic and morphogenic activities in various types of cells via its functional receptor, MET [9,10]. HGF is required for organogenesis in the embryonic stage and for tissue repair in adulthood during organ diseases [5–8]. Several lines of *in vitro* studies have indicated that HGF stimulates the scattering and migration of cancer cells [11–13]. On the other hand, *MET* mutation is causative for familial carcinomas, such as renal carcinoma or head-and-neck carcinoma in humans [14]. In malignant tumors, HGF is produced by stromal cells, while MET is expressed by cancer cells, which suggested in the mid-1990s that this paracrine loop may determine malignant behaviors [11–13].

NK4 is an intra-molecular fragment of HGF, and is composed of an *N*-terminal hairpin domain and 4-kringle domains (K1–K4) of HGF α-chain [15,16]. This fragment also binds to MET, but does not activate the receptor signal transduction. Using NK4 as an HGF-antagonist in experimental cancer models, we demonstrated that endogenous HGF–MET cascade plays a central role in tumor metastasis. In other words, inhibition of the HGF–MET cascade can be a pathogenesis-based approach to the blockage of malignant events. Various candidates have been implicated as HGF-antagonists or MET-inhibitors (such as anti-HGF antibodies and small-sized MET-inhibitor); all are promising for inhibiting malignant behaviors. This review focuses on the roles of endogenous HGF in cancer biology and pathology. We also emphasize the significance of the NK4 discovery for cancer biology and pharmaceutical developments of anti-cancer drugs.

## 2. Discovery of HGF and MET

HGF was discovered as a mitogen of rat hepatocytes [2–4]. Endogenous HGF is essential for protection and repair in numerous organs. The decrease in HGF production leads to organ failures, and HGF supplement therapy is reasonable for the attenuation of organ diseases [8]. Endogenous HGF is also required for organ development in embryogenic stages. On the other hand, MET was identified as a mutant oncogenic protein that induces malignant formation in soft agar [17]. MET has tyrosine kinase activities that are phosphorylated during tumorigenicity, suggesting the presence of ligand-like growth factors. HGF has been identified as a phantom ligand of MET [9,10]. Prior to discussion on the oncogenic roles of HGF, we will postulate the physiological importance of HGF, focusing on organ development and regeneration.

### 2.1. Discovery of HGF and Scatter Factor

**Figure 1:**
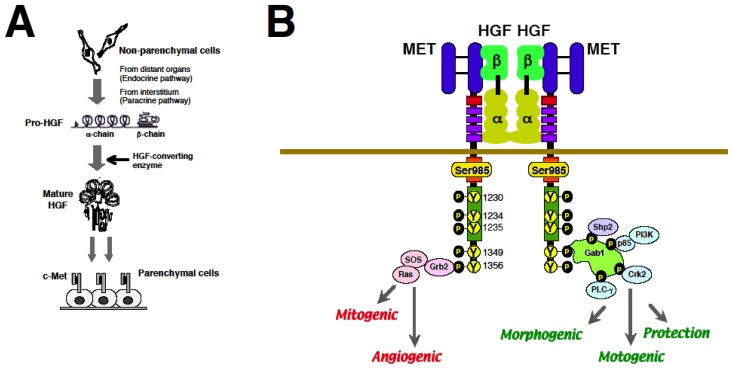
Production and various biological functions of HGF. (**A**) Production, activation and delivery of HGF. HGF is produced by mesenchymal cells, such as fibroblasts. HGF is secreted in an inactive form (*i.e*., pro-HGF). Under diseased conditions, pro-HGF can be converted to mature HGF by HGF-converting enzymes, such as u-PA. HGF is delivered to injured sites via endocrine and paracrine pathways; (**B**) Multiple biological actions of HGF, mediated via MET tyrosine phosphorylation, are outlined. These activities depend on downstream adaptor molecules recruited by MET tyrosine-phosphorylated multi-docking sites. In developing or regenerating tissue, HGF induces MET activation via the formation of a 2:2 complex where MET dimerization is mediated by dimer formation of HGF [6–8].

HGF was identified in 1984 as a long-sought hepato-trophic factor. HGF was purified from serum of rats after 70%-partial hepatectomy [2], and this factor potently stimulated DNA synthesis and proliferation of primary culture of adult rat hepatocytes. In the mid-1980s, we purified HGF to homogeneity from blood platelets of 3000 rats and demonstrated that it was a new growth factor [18]. Based on complete purification of native HGF, cloning of HGF cDNAs was successfully performed, followed by the complete amino acid sequence of human HGF determined from the nucleotide sequences of cDNAs [3,4] (Figure 1A).

Scatter factor (SF) was identified as an embryonic lung fibroblast (MRC5)-derived factor that enhances motility of renal cells (MDCK) [19]. Such SF-induced motogenic activities were abolished by anti-HGF antibody, while recombinant HGF mimicked the scatter activity in the culture of MDCK [20]. Indeed, MRC5-fibroblasts expressed 6 kb mRNA that was hybridized with a HGF cDNA probe, and SF cDNA cloned from the MRC5 cDNA library had the same sequence as HGF of cDNA from human leukocytes [20]. Overall, it was shown that SF is identical to HGF [20,21], suggesting roles of HGF/SF during cancer metastasis.

### 2.2. Identification of MET as a Functional Receptor for HGF

In the mid-1980s, *MET* was identified as a “mutant” oncogenic gene. Dean *et al*. isolated *MET* from carcinogen-induced osteosarcoma cells (MNNG-HOS), which induced NIH-3T3 transformation in soft agar [17]. The *MET* proto-oncogene is localized to the seventh chromosome (7q21–q31) in humans. The *MET*-encoding protein has a tyrosine kinase activity, suggesting that MET is an orphan receptor of growth factors. On the other hand, HGF was identified as a mitogen of hepatocytes [2–4], but in the late 1980s, its receptor was still unknown.

Bottaro *et al*. reported in 1991 that MET may be a signaling receptor for HGF, along with the tyrosine phosphorylations [9]. The protein band was identified as an antigen of anti-MET antibody in the cross-linking of ^125^I-labeled NK1 (an HGF-variant) to cellular protein extraction. High-affinity binding cellular sites specific to HGF were detected in *MET* cDNA-transfected COS7 cells, with a Kd value of 30 pM. Only *MET*-transfected COS7 showed the mitogenic response to HGF, thus indicating that “wild-type” MET is an HGF-receptor [10].

### 2.3. Biological Properties of HGF via MET

In the extra-cellular domain of MET, the first 519 amino aids form a 7-bladed β-propeller domain, called Sema domain, which is necessary for binding to the HGF β-chain and activation of the receptor by ligation. The MET immunoglobulin-like domain is required for binding to the first kringle domain (NK1) of HGF α-chain. HGF induces MET activation via the formation of 2:2 complex where MET–MET dimerization is mediated via dimmer-formed HGF [7,8] (Figure 1B).

The accurate binding of HGF to MET triggers signaling transduction. The ATP-dependent phosphorylation at three residues in the MET active loop kinase domain, Tyr-1230/34/35, is an initial step for activating MET. Phosphorylation at Tyr-1349/56 in the *C*-terminal docking site is required for various bio-functions via recruiting downstream adopters. For example, phospho-Tyr1349/56-dependent recruitment of Grb2-SOS activates Ras-ERK cascades, leading to cell proliferation. Association and tyrosine phosphorylation of Gab-1, a docking protein that couples MET with multiple signaling proteins such as PI-3kinase, PLC-γ, Shp-2, and Crk-2, plays definite roles in HGF-induced morphogenesis and motility. Various adaptor molecules, recruited at MET docking-site, explain multi-faceted functions of HGF [7,8] (Figure 1B).

### 2.4. HGF–MET System for Organ Development and Regeneration

In embryonic organs, HGF is mainly produced in stroma cells such as fibroblasts, while MET is expressed by parenchymal cells [4–8], suggesting that the paracrine loop of HGF–MET is critical for organ development. Indeed, HGF acts as a mesenchyme-derived morphogenic factor during fetal lung development. When HGF-antisense oligo-DNA was added to embryonic lung cultures, stromal HGF production became negligible, followed by impairment in lung branching morphogenesis [22]. *In vivo*, lung alveoli-specific *MET* gene destruction led to a decrease in alveologenesis in mice. Organ-specific *MET* deletion techniques revealed pivotal roles for HGF in development of various organs, such as liver, kidney muscle, *etc.* [6,8].

Endogenous HGF is also important for tissue repair and protection *in vivo*. We provided the first evidence that recombinant HGF enhanced hepatic regeneration in mice with hepatitis [23]. Therapeutic effects of recombinant HGF were also seen in injured organs, such as kidneys, lung, stomach–intestine, skin, heart, brain *etc.* [8]. Blood HGF levels markedly increase in patients and rodents during tissue injuries. When anti-HGF antibody was administered to a rat model of myocardial infarction, cardiac damage was exacerbated [24]. Such a key role of endogenous HGF has been seen in acute and chronic organ diseases [8,25]. Thus, compensation for the loss in intrinsic HGF by HGF administration is a logical strategy to improve organ failures [8,25].

## 3. Roles of HGF–MET Axis in Tumorigenesis

Cancer growth continues beyond the cell–cell contact inhibition system, and cancer has been described as a neverhealing wound. Thus, molecular elucidation of oncogenesis remains to be a central interest for basic scientists. As mentioned, HGF is a key paracrine regulator for embryogenesis and organ regeneration. In contrast, *MET* mutation is responsible for familial renal carcinoma as well as for other sporadic types of cancers [14]. Regardless of the presence or absence of *MET* mutation, stroma-secreted HGF plays a common role in tumor invasive growth. In this section, we will discuss the roles of HGF–MET pathways for cancer onset and development, with an interest in molecular mechanisms.

### 3.1. Roles of MET Mutations for Oncogenesis

#### 3.1.1. *In Vitro* Study

Originally, *MET* was identified as an oncogene that promotes anchorage-independent growth of osteosarcoma cells in soft agar [17]. An initial report described that a mouse homolog of the human *MET* oncogene is amplified 4- to 8-fold in 7 of 10 lines of transformed NIH-3T3 fibroblasts in the soft agar culture. In addition, overexpression of HGF is also capable of inducing malignant phenotypes in soft agar. When rat *HGF* cDNA was introduced into immortalized mouse liver epithelial cells (MLE10), all MLE10-HGF cell lines grew much faster than the original MLE10 cells in culture and produced in large colonies in soft agar [26], suggesting the involvement of aberrant MET signals in tumor onsets.

#### 3.1.2. Animal Study

In the mid-1990s, several groups created the transgenic mice to evaluate the tumorigenic roles of HGF *in vivo*. The MT1-HGF-transgenic mice developed a remarkably broad array of distinct tumors in several tissues [27], including mammary glands, muscles, and skin, which was associated with the over-activation of MET tyrosine kinase. Likewise, local expression of HGF-transgene via an organ-specific gene promoter led to a higher incidence of tumor formation in muscles, airways, skin and mammary glands [27,28]. These transgenic animals are useful as a model to elucidate the tumorigenic mechanisms.

#### 3.1.3. Human Studies

*MET* mutations are causative for human cancers. In patients with papillary renal carcinoma (PRC), missense mutations in the MET tyrosine kinase domain were detected in the germ-line of familial PRC (*i.e*., M1149T, V1206L, V1238I, D1246N and Y1248C) and in a subset of sporadic PRC (*i.e*., L1213V, D1248H and M1268T) [14]. Likewise, somatic mutations in the kinase domain (Y1230C and Y1235D) were identified in head-and-neck squamous carcinoma [29]. Mutations of the MET juxta-membrane domain (*i.e*., R988C and T1010I) are also responsible for familial colon cancer, gastric cancer and small cell lung carcinoma [30]. Moreover, one-point mutation of the MET extra-cellular domain (*i.e*., N375S) was seen in 13% of East Asians with lung cancers [31]. There is now ample evidence showing that *MET* mutations observed in some MET-domain sites cause various types of solid tumors.

### 3.2. Molecular Basis of MET-Mediated Cancer Development

Since constitutive activation of the MET signal is one of the key oncogenic events, it is important to discuss its molecular basis, focusing on downstream MET. Using some mutations of *MET* identified in patients with familial PRC, Giordano *et al.* found that some mutated *MET* enhance the Ras signaling pathway [32]. Other mutations are devoid of transforming potential but are effective in inducing protection from apoptosis, associated with the efficient interaction of PI3Kinase. Thus, different mutations in the *MET* gene may elicit tumorigenesis via Ras-based mitogenesis and PI3Kinase-based protection pathways [32].

β-catenin is an oncogenic protein involved in the regulation of cell–cell adhesion and gene transcription. Once β-catenin is activated, it is translocated from sub-membrane to nucleus, and then β-catenin functions as an activator of Tcf-transcriptional factor, leading to upregulation of mitogenic proteins, such as c-Myc and cyclin-D1. HGF activates β-catenin via a direct assembly of phospho-MET and β-catenin. Likewise, active *MET* mutants (such as M1268T) also activate the nuclear β-catenin function [33]. Thus, aberrant activation of nuclear β-catenin by active MET leads to tumor formation via the upregulation of c-Myc and cyclin-D1.

Src kinase activity is important for mutant *MET* to acquire tumorigenic potentials. In the NIH-3T3 clone (F4) that overexpresses the mutant *MET* (M1268T), malignant formation was evident in both soft agar and implanted nude mice [34]. These malignant phenotypes were abolished by dominant negative c-Src in the NIH-3T3-F4 clone. A recent report described how the direct assembly of MET by a membrane Fas-ligand is also required for tumorigenic potentials, possibly via sequential phosphorylations from MET to the downstream Stat3, a key regulator for enhancing anchorage independent growth [35].

MET overexpression and its crosstalk with other receptors, such as EGFR, Her2, Her3 are also critical for tumor development. EGFR tyrosine kinase inhibitors (EGF-TKI) intercept EGFR-mediated downstream signaling pathways (such as PI3K-AKT) via de-phosphorylation of Her3, while some lung carcinoma cell lines acquire resistance via amplification of MET [36]. In this process, amplified MET trans-phosphorylates Her3, and then impaired AKT pathways are restored, resulting in resistance to EGFR-TKIs, including Gefitinib (Iressa^®^) [36,37]. Such a redundant signaling via amplified MET may participate in breast cancer malignant behaviors during the treatment with anti-Her-2 antibody, Trastuzumab (Herceptin^®^) [38], possibly due to association (and re-activation) of Her2 with amplified MET [39,40]. Thus, MET inhibition can be a target to block the redundant pathways, required for drug resistance (see Section 6.4).

In summary, several lines of human and animal studies have demonstrated that not only *MET* mutations, but also HGF over-productions, lead to oncogenenic outcomes via over-activation of *MET*-associated molecular pathways, such as Ras, β-catenin *etc*. (Table 1).

## 4. HGF-Mediated Cancer Metastasis

Oncogenic *MET*-mutants are responsible for PRCs [14] and head-and-neck carcinoma [29], but these are not common in many types of malignant tumors. In contrast, HGF plays common roles in cancer metastasis [13,58], independent of the presence or absence of *MET* mutations. This may be one of the reasons why HGF-antagonistic treatment is a logical approach to induce invasion-free conditions, like benign tumors. Prior to referring to the successful results of NK4 in rodents, the mechanisms of HGF-mediated tumor metastasis will be discussed.

### 4.1. Functions of HGF for Tumor Cell Scattering and Invasion

Tumor cell scattering in the primary tumors is the first step for metastasis. In tumor tissues, stroma-secreted HGF is required for cancer cells to infiltrate neighboring tissues, such as vascular beds, across the basement membrane. The paracrine loop between HGF-producing stroma and MET-expressing tumor cells acts as a local switch for the invasive phenotypes, as follows.

#### 4.1.1. Tumor Cell Dissociation

The initial events for the metastatic spread involve loss of cell–cell contact within primary tumors. The integrity of epithelial tumor cell colonies is maintained by cell–cell contact mediated by cadherins and intracellular catenin molecules. Cancer cells must lose their tight cell-to-cell contact by downregulation of cadherin–cadherin complex during invasion into adjacent tissues. HGF induces dispersion of cluster cells into single cells via an endocytosis of E-cadherin from cell surface to cytoplasma [41,42].

#### 4.1.2. Cell Movement

During tumor cell motility, HGF stimulates the Ras-Rab5 cascade for initiating endocytosis of cadherins [42], followed by nuclear localization of β-catenin, a transcription factor of genes required for cell motility [43]. HGF-mediated stimulation of Rho small G protein cascade and activation of cdc42/Rac/PAK are required for disassembly of stress fiber or focal adhesions, and then lamellipodia formation and cell spreading are enhanced [44]. Such sequential downstream events of MET signaling are contributable for cancer cell motility.

#### 4.1.3. Basement Membrane Breakdown

For adjacent invasion, tumor cells must move across a basement membrane between the epithelium and submucosa. Cancer cells spread rapidly and form focal adhesions, and then they disassemble these condensations, followed by enhanced cell locomotion. In the initial phase, HGF induces phosphorylation of focal adhesion kinase (FAK) together with a bridge between the ECM and integrins of cancer cells [11,45]. In a later phase, HGF upregulates several types of MMP, such as MMP-1/-9/-14 via the activation of Ets [46–48]. HGF-mediated invasion is abolished by MMP inhibitors. Overall, in addition to cell motility, the induction of MMP via HGF-Ets pathways is required for cancer invasion.

#### 4.1.4. Hypoxia as a Trigger for Invasion

Tumor hypoxia occurs in the central areas of a growing mass, due to an insufficient blood flow. Such local hypoxia triggers tumor invasion via upregulating hypoxia-induced factor-1 (HIF1), a transcriptional factor that regulates several genes of cell motility, possibly in order to escape from hypoxic milieu. For this purpose, tumor cells acquire MET via the HIF-dependent pathway, while stroma-secreted HGF enhances cell motility from hypoxic regions to the aerobic milieu of distant organs (*i.e*., successful metastasis) [59,60]. The HIF1-MET pathway may underlie a key mechanism of hypoxia-initiated malignancies.

### 4.2. Roles of HGF during Metastatic Cascade

In addition to adjacent invasion, the HGF–MET axis is important for distant metastasis, through extravasation, anti-anoikis and homing. HGF promotes cancer metastasis via inducing vascular bed formation and enhancing chemokine-induced homing. HGF also protects cancer cells from anoikis or immunological challenge. These sequential events lead to successful metastasis.

#### 4.2.1. Angiogenic Events

Vascular angiogenesis is a critical step for the delivery of cancer cells from the primary tumors to secondary organs. HGF enhances angiogenesis via the induction of growth, movement and morphogenesis of endothelial cells (EC) in a culture system [53,61] and in a mouse model of glioma [62]. HGF has a direct effect on EC via the enhancement of cancer cell-EC contact via FAK phosphorylation [55]. HGF decreases endothelial occludin, a cell–cell adhesion molecule [56]. As a result, HGF decreases the trans-endothelial resistance of tumor vessels, and then cancer invasion across an EC barrier (*i.e*., intra-vasation) is achieved.

#### 4.2.2. Anti-Anoikis

Suspension-induced apoptosis, also known as anoikis, is defined as apoptosis of parenchymal cells that is induced by loss of ECM attachment. Cancer cell resistance to anoikis (*i.e*., anti-anoikis) is important for achieving metastasis, because tumor cells lose matrix attachment during blood flow. HGF inhibits suspension-induced anoikis, and this effect is mediated by PI3-kinase and ERK-1/2 [49]. Inhibitory effect of HGF on tumor anoikis is abolished by tetraspanin CD151 deletion [50]. Thus, signaling complexes between MET and integrin-tetraspanin are needed for anti-anoikis, together with an involvement of MET/PI3-kinase/ERK cascades.

#### 4.2.3. Homing

Stroma-derived factor-1 (SDF1) is a chemokine that induces cancer metastasis. Distant organs produce SDF1, whereas invasive cancer cells acquire CXCR4, a receptor of SDF1. Such an endocrine pathway is required for moving cells to determine the homing sites. Of note, HGF enhances CXCR4 expression by cancer cells [51]. Furthermore, HGF enhances the ability of SDF1 to promote cancer invasion [52]. Thus, a network between the HGF–MET axis and the SDF1-CXCR4 axis seems to be essential for determining the homing sites.

#### 4.2.4. Immune Tolerance

A host immune surveillance system is important in the control of tumor metastasis. Dendritic cells (DC) play a key role in an immunological challenge, via recruiting cytotoxic T cells. HGF suppresses immune surveillance via the induction of anti-inflammatory cytokines, such as IL-10, in DC [57]. Such HGF-mediated modification of DC (*i.e*., tolerogenic DC) leads to an escape of cancer cells from immunological challenges, as suggested [63].

### 4.3. Regulation of HGF Production during Cancer Progression

In addition to oncogenic mutants, the stromal environment plays a key role in cancer prognosis. Carcinoma-associated fibroblasts (CAF), but not normal fibroblasts, promote tumor progression of initiated non-tumorigenic epithelial cells. Stroma-derived HGF is responsible for tumor invasive growth [11–13]. Thus, the molecular basis of HGF production should be discussed.

Numerous types of carcinoma cells secrete soluble factors that induce HGF production in stromal cells (*i.e*., HGF-inducers). For example, conditioned medium of breast cancer cells enhances HGF production in fibroblasts, along with a raise in prostaglandin-E2 [64]. Inhibition of prostaglandin-E synthesis by indomethacin leads to downregulation of HGF production and suppression of tumor migration, indicating that cancer-derived prostaglandins are important for stromal HGF production [64]. Other cancer-derived HGF-inducers are IL-1β, b-FGF, PDGF, and TGF-α [12,65]. Such a paracrine loop, mediated by the cancer-derived HGF-inducers and stroma-secreted HGF, confers a key mechanism of tumor metastasis [66].

In addition to CAF, tumor-associated macrophages (TAM) are a major source of HGF in tumor tissues. TAM isolated from 98 primary lung cancer tissues shows the higher ability to produce HGF. This is also the case for HGF produced by neutrophils infiltrating bronchiolo-alveolar subtype pulmonary adenocarcinoma [67]. Serum levels of HGF are elevated in patients with recurrent malignant tumors [68], thus suggesting an endocrine HGF delivery system. In this regard, peripheral blood monocytes are known to produce HGF, contributing to the increase in blood HGF levels via an endocrine mechanism [69].

In summary, stroma-derived HGF is required for cancer invasion (*i.e.*, paracrine system). Moreover, blood leukocytes inhibit anoikis and enhance SDF1 signaling in tumor cells during blood flow via a secretion of HGF into blood (*i.e*., endocrine system). The roles of HGF during tumor growth (see Section 3) and metastasis are summarized in Table 1.

## 5. Preparation of NK4 as HGF-Antagonist

HGF is a stromal-derived factor that stimulates cancer invasion at least *in vitro* [11–13,66]. The degree of serum HGF and MET expressions in cancer tissues correlates with the prognosis of patients [68]. Even in cases of *MET* mutation, HGF is important for tumor malignancy [58]. Thus, inhibition of HGF–MET signaling may be a logical strategy to prohibit cancer metastasis. To test this hypothesis, NK4 was prepared as an intra-molecular fragment of HGF. As expected, NK4 binds to MET and intercepts HGF–MET coupling as a competitive inhibitor. Strikingly, NK4 inhibits tumor angiogenesis through a MET-independent pathway. This section focuses on the dual role of NK4 as an HGF-antagonist and as an angiogenesis-inhibitor.

### 5.1. Structure and Anti-Invasive Function of NK4

**Figure 2:**
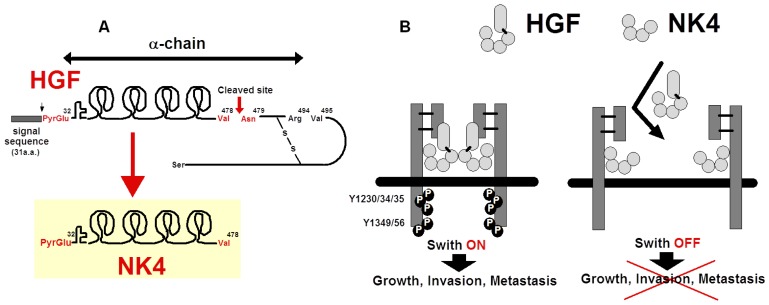
Preparation of NK4 as an HGF-antagonist. (**A**) Preparation and structure of NK4. NK4 is generated via a cleavage of HGF between 478th Val and 479th Asn [15]; (**B**) Inhibition of HGF-mediated MET tyrosine phosphorylation by NK4. NK4 alone binds to MET, but does not phosphorylate MET tyrosine kinases. Thus, the NK4–MET complex inhibits HGF–MET interaction via a masking of the HGF-binding motif in MET domains.

NK4 was initially purified as a fragment from elastase-digested HGF samples [15,70]. The amino acid sequence revealed that NK4 is cleaved between Val^478^ and Asn^479^ by elastase. The *N*-terminal structure of NK4 is the same as undigested HGF (*i.e*., 32nd pyroglutamate), indicating that NK4 is composed of the *N*-terminal hairpin domain and 4-kringle domains (thus designated NK4) (Figure 2A). Precursor HGF is cleaved between Arg^494^ and Val^495^, while NK4 is cleaved between Val^478^ and Asn^479^. In other words, NK4 is identical to HGF α-chain that lacks *C*-terminal 16 amino acids (*i.e*., Asn^479^ to Arg^494^) (Figure 2A). The domains that are responsible for high-affinity binding to *MET* are the *N*-terminal hairpin and the K1 domains in NK4.

Although NK4 is a MET-binder, NK4 alone does not phosphorylate MET tyrosine residues, indicating that NK4 acts as a complete HGF-antagonist (Figure 2B). Actually, MET tyrosine phosphorylation occurs in A549 lung carcinoma cells within 10 min after HGF addition, while NK4 inhibits the HGF-mediated MET activation. HGF induces invasion and migration of the gallbladder cancer cells in Matri-gels, while NK4 inhibits HGF-induced invasion [15]. These anti-invasive effects of NK4 are also seen in distinct types of cancer cells, strengthening the common role of NK4 in cancer migration [66].

Other HGF fragments, NK1 (composed of hairpin-loop and K1 domain) and NK2 (hairpin-loop and K1–K2 domains), are natural variant forms of HGF. NK1 is often agonistic to MET, particularly in the presence of heparin, but it sometimes elicits antagonistic effects. NK2 is antagonistic but sometimes agonistic to HGF–MET signaling. Indeed, forced expression of NK2 transgene leads to the acceleration of metastasis in mice bearing melanoma [71]. In contrast to these variants, NK4 is a complete antagonist (*i.e*., without agonistic features). Therefore, this fragment is reasonable as a practical tool for anti-metastatic strategies (see, Section 6).

### 5.2. Perlecan-Dependent Anti-Angiogenic Mechanisms of NK4

Vascular EC expresses MET, and HGF stimulates mitogenic and morphogenic activities in EC [4,61]. Thus, it is possible that NK4 inhibits HGF-induced angiogenesis. Expectedly, NK4 potently inhibited the HGF-mediated proliferation of EC *in vitro* [72]. Strikingly, NK4 also inhibited EC proliferation, induced by other angiogenic factors, such as b-FGF and VEGF [54]. In this process, HGF and VEGF phosphorylate MET and KDR/VEGF-receptor, respectively, whereas NK4 inhibits HGF-induced MET tyrosine phosphorylation, but not KDR activation [54]. Nevertheless, NK4 inhibits VEGF-induced proliferation, without modification of VEGF-mediated ERK1/2 activation. The same effect of NK4 was also seen *in vivo*: b-FGF-induced angiogenesis in the rabbit cornea was inhibited by NK4. Thus, another mechanism (*i.e.*, MET-independent pathway) may be involved in NK4-mediated arrest of angiogenesis. This alternative pathway is demonstrated in 2009, as follows.

**Figure 3:**
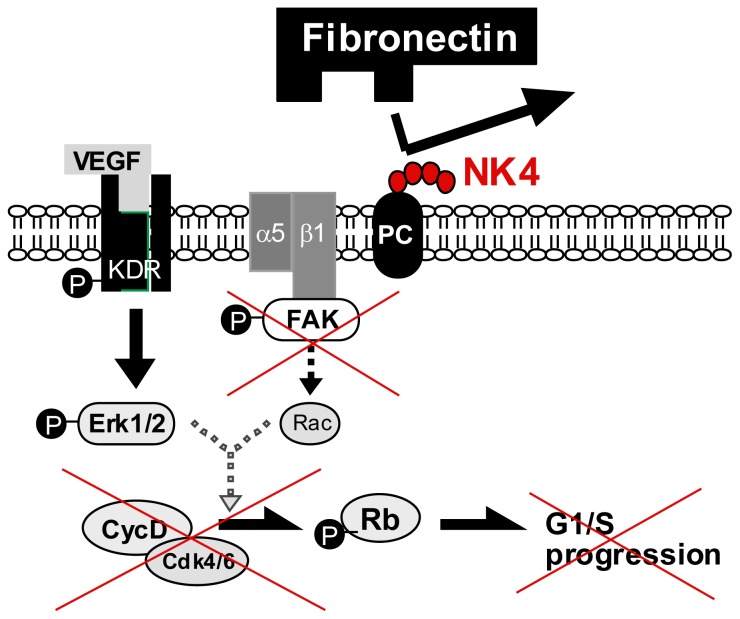
Involvement of perlecan (PC) in NK4-mediated growth arrest of endothelial cells. Cell surface PC is required for the binding of fibronectin and α5β1-integrin, leading to FAK phosphorylation and crosstalk of VEGF–VEGF receptor (KDR) signaling. NK4 binds to PC, and then the binding of fibronectin to integrin is impaired. As a result, VEGF fails to elicit G1/S progression of endothelial cells in the presence of NK4 [54,73].

The fibronectin-integrin signal in EC is required for angiogenesis. The NK4-mediated growth arrest of EC is due to a loss of the fibronectin-integrin signal [73]. The affinity purification with NK4-immobilized beads revealed that perlecan is a counterpart to trap NK4. Perlecan is a heparan sulfate proteoglycan that interacts with basement membrane ECM, such as fibronectin. Deletion of perlecan expression by siRNA diminished the fibronectin assembly and EC spreading, indicating a key role of fibronectin-perlecan interaction during EC migration. Notably, NK4-perlecan interaction reduced the assembly of fibronectin by perlecan. As a result, FAK activation became faint due to the inhibition of perlecan-fibronectin complex with NK4. Under such a loss of integrin signals, EC growth was impaired [73]. This MET-independent pathway is involved in the inhibitory effect of NK4 on VEGF-mediated angiogenesis (Figure 3).

### 5.3. Possible Cleavage of NK4-Like Fragment *in Vivo*

In addition to pancreatic protease, endogenous proteases can generate an NK4-like fragment. Leucocyte-derived proteases, such as mast cell chymase, are able to convert HGF to fragments, including NK4 and remnant HGF-β [74,75]. Endogenous cathepsin-G and MMP also produce NK4-like fragment via its enzymatic reaction, suggesting that *de novo* synthesized NK4 may have a counteractive role in inhibiting HGF-mediated functions under pathological conditions.

For example, NK4-like fragments have been detected in the effusive fluid from skins with intractable ulcers in patients [75]. In this process, kallikrein, an endogenous serine-protease, is critical for HGF-to-NK4 conversion at the local sites. Endogenous HGF is required for skin regeneration [8]. Thus, it is likely that the synthesis of HGF-antagonistic NK4, in addition to the decrease in HGF (*i.e*., intrinsic repair factor), underlies the key mechanism whereby epidermal regeneration is impaired in the patients with intractable leg ulcers [75].

During tumor progression, cancer cells may produce NK4 for tumor growth retardation. Indeed, NK4-like fragments are detectable in the culture supernatants of certain tumor cell lines that produce HGF [76,77]. Although it is still unclear whether NK4-like fragment is generated via an enzymatic cleavage or variant transcription, NK4-like fragment(s) could act as a natural HGF-antagonist to minimize tumor metastasis (as a negative feedback). Thus, exogenous NK4 supplementation may be a reasonable approach to arresting tumor malignancy, as follows.

## 6. Anti-Cancer Strategy by NK4

Given that tumor-stroma interaction via HGF–MET axis is involved in cancer metastasis, inhibition of MET signaling may be a pathogenesis-based anti-tumor strategy. In the late 1990s, several oncologists proposed a proof of concept that tumor angiogenesis inhibition produces anti-metastatic results [78]. NK4 also produces anti-angiogenic effects via a perlecan-dependent pathway. Thus, the dual effects of NK4 (*i.e*., HGF-antagonist and angiogenesis-inhibitor) could provide a new concept whereby NK4 may prove therapeutic for malignant tumors.

### 6.1. First Evidence of HGF-Antagonist for Inhibition of Tumor Progression in Vivo

HGF, or co-cultured fibroblasts, induces invasion of gallbladder carcinoma cells (GB-d1) across Matrigel, an experimental mimic of the basement membrane [46]. NK4 competitively inhibits the binding of HGF to MET on GB-d1 cells. As a result, NK4 diminishes HGF-induced, or fibroblast-induced, motogenic activities [16]. Such a major role of HGF was also seen *in vivo*. Subcutaneous inoculations of GB-d1 cells in nude mice allow for primary tumor growth and invasion to adjacent muscular tissues. Using NK4 in this model, we provided the first evidence that HGF- antagonist can arrest tumor invasion *in vivo* [16]: recombinant NK4 inhibited the growth and muscular invasion in mice bearing GB-d1 carcinoma. Consistent with growth arrest, apoptotic change was evident in NK4-treated mice. This is the first proof of concept to show that HGF-antagonist is useful for anti-tumor therapy.

### 6.2. Inhibition of Tumor Angiogenesis by NK4 Treatment

In culture of EC, NK4 produces anti-angiogenetic effects via a MET-independent pathway, as reported [54,73]. These effects are also observed in animal models of malignant tumors: recombinant NK4 suppressed the primary tumor growth, metastasis of Lewis lung carcinoma, and Jyg-MC(A) mammary carcinoma in mice [54], although neither HGF nor NK4 affected proliferation and survival of these tumor cells *in vitro*. NK4 treatment resulted in a remarkable decrease in vessel density and an increase in apoptotic cells in the tumor tissues [54]. NK4-induced anti-angiogenic effects are reproduced in various types of cancers [79–81]. Since inhibition of angiogenesis by NK4 results in tumor hypoxia, hypoxia-primed apoptosis may be involved in cancer growth arrest by NK4.

### 6.3. Delayed NK4 Therapy for Attenuation of End-Stage Carcinoma

**Figure 4:**
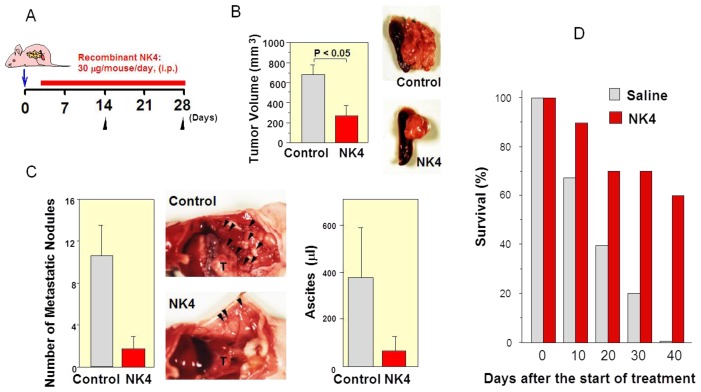
Anti-metastatic effects of NK4 on advanced pancreas cancer in mice. (**A**) Schedules for NK4 treatment of mice with pancreatic cancer. NK4 was injected into mice between 3 and 28 days after the inoculation of pancreatic cancer cells (SUIT-2); (**B**) Inhibition of primary tumor growth by NK4. Photographs show appearance of the primary pancreatic cancers; (**C**) Inhibitory effects of NK4 on peritoneal metastasis. **Left**: Changes in the number of metastatic nodules. Middle: Macroscopic findings of metastasis. **Right**: Changes in the ascite volumes; (**D**) Prolonged survival of mice treated with NK4.

Anti-tumor effects of NK4 is also observed in advanced carcinoma with metastasis [79]. When NK4 treatment was initiated on day 10 (a time when cancer cells were already invading surrounding tissues), NK4 potently inhibited the tumor growth, peritoneal dissemination, and ascites accumulation at four weeks after the tumor inoculation. As a result, NK4 prolonged the survival time of mice at an end-stage of cancer (Figure 4). Because effective systemic therapy for pancreatic cancer is currently not available, and diagnosing pancreatic cancer in its early stages is difficult, the highly invasive and metastatic behaviors lead to difficulty in attaining a recurrence-free status. Blockade of HGF-mediated invasion may prove to be potential therapy for advanced stages of pancreatic carcinoma.

### 6.4. Therapy Combining NK4 with Other Treatments

Chemotherapy, radiation therapy and anti-angiogenic treatment are applicable in patients with inoperable tumors. Recent reports demonstrated the synergic anti-tumor effect of NK4 on these conventional therapies in rodents, as follows.

#### 6.4.1. Chemotherapy

**Figure 5:**
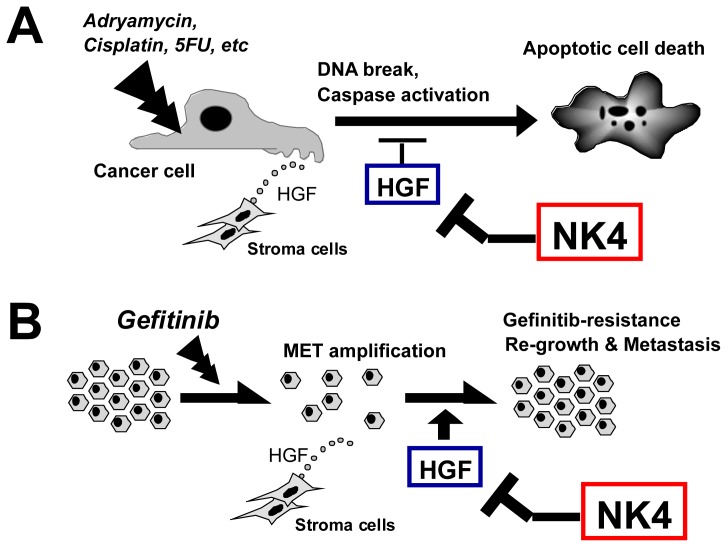
Release of HGF-mediated drug resistance by NK4 in cancer cells. (**A**) NK4 releases the HGF-mediated protection of cancer cells from DNA-damaging agents, such as Adriamycin and cisplatin. HGF prevents DNA single-strand breaks via the rapid induction of DNA repair. In addition, HGF inhibits caspase-3 activation and prohibits apoptosis. NK4 can antagonize the HGF-mediated protections of cancer cells; (**B**) EGF-receptor TK inhibitors, such as Gefitinib, induce cell death in an early phase, but cancer cells acquire drug resistance via MET gene amplification or HGF-dependent pathway [36,85]. NK4 restores the loss in Gefitinib sensitivity by the counteraction of HGF actions as an HGF-antagonist.

HGF protects cancer cells from chemo drugs, such as Adriamycin, cisplatin and 5FU via the different mechanisms. One is that HGF enhances DNA repair from drug-induced single strand DNA breakage [82]. Another is that HGF elicits anti-apoptotic effects via inducing anti-apoptotic molecules (*i.e.*, Bcl-xL) and reducing caspase-3 activation [83]. Indeed, NK4 enhances cisplatin-induced tumoricidal effects in mouse models [84], perhaps via the release of HGF-mediated protection (Figure 5A). EGF-receptor tyrosine kinase (TK) inhibitors, such as Gefitinib, are known to reduce lung carcinoma metastasis, but long-term use of Gefitinib leads to drug resistance via *MET* gene amplification [36] or HGF-dependent pathway [85]. Thus, NK4 is promising for the release of the Gefitinib resistance as an HGF-antagonist (Figure 5B).

#### 6.4.2. Radiation Therapy

Irradiation often enhances metastasis, particularly in cases of pancreatic carcinoma, and this is associated with the radiation-induced upregulation of MET in cancer cells [86]. HGF enhances the repair of DNA strand breaks caused by γ-radiation [82]. Radiation can also enhance the secretion of HGF from malignant glioma [87]. HGF also protects ECs from radiation-induced apoptosis [88]. Thus, NK4 likely enhances radiation therapy via counteracting HGF-mediated protection from radio-toxicity.

#### 6.4.3. Immune Therapy

A recent study demonstrated that NK4-mediated tumor regression depends on cytotoxic T lymphocytes (CTL) [63]. NK4 treatment reduced the tumor growth and invasion in a mouse model of colon cancer, and this was associated with the enhanced infiltration of CD8+ CTL. Of note, such an anti-tumor activity of NK4 was abolished by depletion of CD8+ cells in this mouse model. Taken together, NK4 may also have utility for anti-tumor immunotherapy.

#### 6.4.4. Anti-Angiogenic Therapy

**Figure 6:**
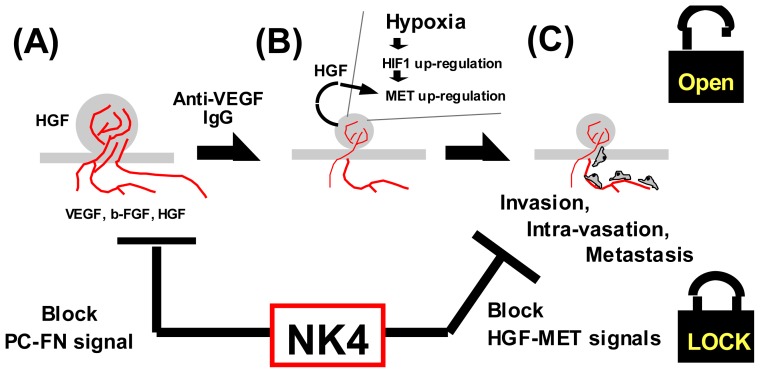
Freeze and dormant therapy of malignant tumors by NK4, an HGF-antagonist and angio-inhibitory agent. (**A**) Primary tumors show invasive growth under the support of vascular formation, mediated by VEGF, b-FGF and HGF. Stroma-secreted HGF also supports tumor growth and invasion via activations of Ras and β-catenin pathways; (**B**) Anti-angiogenic strategies, using anti-VEGF antibody, lead to tumor hypoxia and regression in a short period. The local hypoxia upregulates MET via HIF1-mediated cascades; (**C**) Under such a hypoxic condition, MET-expressing cancer cells migrate to adjacent vessels in response to HGF. Thus, NK4 is reasonable for suppressing hypoxia-mediated tumor progression: (i) in the early-stage, NK4 reduces tumor angiogenesis via inhibiting binding of fibronectin (FN) to perlecan (PC); and (ii) in the late-stage, NK4 blocks the HGF-mediated cancer invasion and metastasis as an HGF-antagonist. Such a dual property of NK4 produces “freeze and dormancy” therapy against tumor metastasis.

Tumor angiogenesis inhibition is promising as an anti-metastatic strategy [78]. Indeed, anti-VEGF antibody reduces tumor metastasis in the short-term, but long-term treatment often leads to resistance to VEGF antagonism [89]. Under such a hypoxic state, tumor cells acquire MET via HIF1-dependent cascades [59,60], while local HGF protects these cells from hypoxia-induced apoptosis. HGF–MET signal is also important for hypoxia-triggered metastasis (see Section 4.1) [59]. As a result, cancer cells can invade from hypoxic to aerobic tissues through HGF-mediated motogenic actions. Even if NK4 enhances tumor hypoxia via the inhibition of tumor angiogenesis, hypoxia-mediated metastasis will be avoidable, because NK4 can counteract HGF-mediated metastatic events (see Section 5.1). Such a dual function of NK4 will contribute to tumor freeze and dormancy therapies (Figure 6).

#### 6.4.5. Anti-Cancer Stem Cell (CSC) Therapy

Recent studies delineate emerging roles of CSC in tumor malignancy. In tumor tissues, MET activation is required for sustaining CSC via inducing reprogramming transcription factors (such as Nanog, Sox2, Klf4, Oct4 and c-Myc) [90]. HGF–MET cascade also participates in anti-tumor drug resistance, possibly through CSC conversion [91]. Thus, inhibition of aberrant MET activation holds promise for the eradication of CSC, a key determinant for cancer recurrence after chemotherapy. The potential application of NK4 in the CSC growth prevention warrants further attention.

More than 30 reports demonstrate that NK4 is useful for the inhibition of growth, invasion and metastasis in various types of cancers (Table 2) [92]. These data support the hypothesis that HGF is a key paracrine regulator that is responsible for tumor malignancy [11–13].

## 7. Other Anti-Cancer Strategies for Blocking HGF–MET Signaling

Using NK4 in a mouse model of carcinoma, we firstly found in 1998 that MET inhibition by an HGF-antagonist inhibits tumor invasive growth *in vivo*. In the following year, another group reported that deletion of *HGF*, or of *MET*, from glioma cells via a ribozyme technique led to growth arrest in nude mice [104]. Like NK4, anti-HGF antibody suppresses tumor progression in rodents [79,105]. There are now several approaches to blockage of the HGF–MET pathway.

### 7.1. HGF-Mimic Molecules or HGF-Fragments

#### 7.1.1. Pro-HGF

HGF is secreted from stromal cells in a form of precursor (*i.e*., pro-HGF). For conversion to active-form HGF, pro-HGF is cleaved at Arg^494^ and Val^495^. A point mutation at this cleaved site leads to the generation of uncleavable pro-HGF [106]. Like NK4, pro-HGF inhibits HGF-mediated MET tyrosine phosphorylations *in vitro*. The systemic expression of pro-HGF suppresses tumor growth and prevents metastatic dissemination in mice [106]. Thus, proteolytic activation of pro-HGF is an essential step in tumor progression, while the inhibition of pro-HGF activation could be an alternative target for controlling tumor metastasis.

#### 7.1.2. Engineering for HGF Mutation

Artificially mutated *HGF*-coding protein is available as an HGF-antagonist. The *N*-terminal active pocket present in HGF β-chain is required for the assembly of MET, while one-point mutation near the pocket regions produces a complete HGF-antagonist. Indeed, each *HGF*-mutant proteins (D672N, V495G, V495A, G498I, and G498V) bind to MET, without any MET activation [107]. These mutants clearly suppress HGF-induced cancer cell migration via the inhibition of MET tyrosine phosphorylation. Conversion of HGF from agonist to antagonist is achieved by as little as removal of two methyl groups (V495A) or a single charge (D672N).

#### 7.1.3. NK1 and NK2

*HGF* variants, NK1 and NK2, show bivalent activities. NK1 is basically agonistic to MET in the presence of heparin. A recent report suggests the potential use of mutant *NK1*-coding protein as an antagonist: HGF induced cell scattering in a culture of MDCK cells, while mutant NK1 (D127K) inhibits the HGF-mediated cell dissociation [108]. It remains controversial as to whether or not NK2 is antagonistic *in vivo*. Toxin-induced hepatitis becomes severer in NK2-transgenic mice, suggesting an antagonistic effect of NK2 (*i.e*., release of HGF-mediated hepatic protection) [109]. In contrast, NK2 transgene accelerated the metastasis in a mouse model of malignant melanoma [71], implying the possible agonistic actions of NK2.

### 7.2. Antibodies to HGF or MET

We provided evidence in 2001 that not only NK4 but also anti-HGF antibody is useful for improving the prognosis of malignant tumors, using pancreatic cancer-bearing mice [79]. In the same year, another group also demonstrated the anti-tumor effect of anti-HGF antibody in a xeno-graft model [105]. These studies stimulated the pharmaceutical companies to develop several types of antibodies specific to HGF or its receptor, MET, as follows.

#### 7.2.1. Anti-HGF Antibodies

Some clones of monoclonal antibodies against HGF were screened *in vitro* and anti-tumor efficacy was evaluated in human cancer-bearing nude mice. Human-type monoclonal antibody against human HGF (AMG102, Rilotumumab, Amgen, Thousand Oaks, CA, USA) was useful for several types of solid tumors. Indeed, AMG102 suppressed the subcutaneous growth of glioblastoma (U-87) in mice, through enhanced apoptosis and lowered mitogenesis [110]. In this model, AMG102 did not modify the tumor angiogenesis. With regard to this, AMG102 prohibited the HGF-induced growth and survival of human endothelial cells (HUVEC) *in vitro*, but VEGF- and b-FGF-induced proliferation of HUVEC were not altered by AMG102 [110], implying the selective effects of AMG102 on HGF-mediated tumorigenicity. Clinical studies of AMG102 are now ongoing, and its safety and efficacy will be carefully evaluated.

In contrast to anti-HGF antibody, NK4 inhibits not only invasive growth, but also tumor angiogenesis (as a perlecan-binder). This may be the reason why NK4 is more efficient than anti-HGF antibody in rodent models [79].

#### 7.2.2. Anti-MET Antibody

The masking of surface MET by specific antibodies is also promising. For instance, MetMab (Genentech, USA) is a single Fab-armed monoclonal antibody, which competitively blocks HGF–MET binding and subsequent cascade activation. Infusion of MetMab into U87-glioblastoma inhibited its growth in mice via enhancing apoptosis [111]. The anti-tumor effect of MetMab was also shown in an orthotopic model of pancreatic cancer. Its safety and efficacy are now being evaluated in subjects with non-small cell lung carcinoma. Other types of anti-MET antibody are also promising. DN30 is an antibody that stimulates MET ectodomain shedding and cleavage of the intra-cellular domain. Injection of DN30 attenuated the metastatic spread to the lung in a mouse model [112]. The LMH80 antibody recognizes an epitope in small cysteine-rich domain of MET [113]. This antibody specifically binds to MET precursor (pro-MET). Considering that pro-MET is expressed on the surface of cancer cells, but not normal cells, this antibody is potentially tumor-specific [113]. Anti-MET antibody is believed to be bivalent (*i.e*., antagonistic and agonistic effects), but the technology of monovalent antibody (*i.e*., antagonistic effect only) may support the anti-tumor therapy.

### 7.3. Small-Sized MET TK Inhibitors

Small molecule MET tyrosine kinase (TK) inhibitors may be practical in a clinical setting, due to an oral administration. Binding of HGF to MET induces a dimer–dimer complex (2:2) and phosphorylates kinase domain active loop (*i.e*., Y1230/34/35) via recruiting ATP. These kinase domain activations rapidly elicit phosphorylation of multi-docking sites (*i.e.*, Y1349/56), and then various activities are producible via recruiting downstream adapter, Grab2 [114]. Thus, blockade of this trans-phosphorylation can be an anti-cancer strategy, as follows.

PHA-665752 was identified as a small-molecule, ATP-competitive inhibitor of the catalytic activity of the MET kinase domain loop (*i.e*., Y1230/34/35). PHA665752 reduced NCI-H69 (small-cell lung cancer) and NCI-H441 (non-small-cell lung cancer) tumorigenicity in mouse xenografts by 99% and 75%, respectively [115]. PHA665752 inhibited MET phosphorylation and c-Cbl binding sites in mouse xenografts derived from non-small cell lung cancer cells (NCI-H441 and A549) and small cell lung cancer cells (NCI-H69) [115].

ATP-competitive inhibitors may suppress MET mutation-induced (*i.e*., HGF-independent) auto-phosphorylation by counteracting the kinase domain (Y1230/34/35) activation. However, some MET mutants are not sensitive to ATP-competitive inhibitors. Under such a resistant state, the MET multi-docking region may be a downstream target. For example, MK-2461, a novel multi-targeted TK inhibitor, suppresses phosphorylation of Y1349, but not Y1234/35 [116]. As a result, MK-2461 reduced the malignant phenotypes in mice bearing *MET*-mutated tumors.

Collectively, not only targeting the kinase domain with ATP-competitive inhibitors but also inhibiting multi-docking sites may provide a therapeutic approach. Although small-sized TK inhibitors facilitate an oral administration (*i.e*., systemic exposure), an effective dose might impair non-tumor normal organs, since endogenous HGF is essential for organ homeostasis [8]. Clinical trials of MET TK inhibitors (such as PHA-665752 or SU-11274) are now being developed [117]. The tumor-specific delivery of these drugs warrants ongoing attention.

### 7.4. Decoys or MET Binders

Decorin is a member of the small leucine-rich proteoglycan gene family and it can impede tumor growth, but its molecular basis has not been elucidated. A recent study identified decorin as a MET antagonist. Decorin binds to MET and phosphorylates tyrosine sites in the kinase domain (*i.e*., Y1234/35) in Hela-cells within 15 min. post-addition [118]. Nevertheless, decorin does not phosphorylate the MET multi-docking site, Y1349. As a result, decorin inhibited tumor growth *in vivo*, probably via the inhibition of the β-catenin pathway.

Norleual, an angiotensin-IV analog, exhibits a structural homology with the hinge region of HGF. Norleual competitively inhibited the binding of HGF to MET in mouse liver membranes, with an IC_50_ value of 3 pM [119]. Predictably, norleual inhibited HGF-dependent signaling, proliferation, migration and invasion in the cultures of multiple cell types at concentrations within the picomolar range. *In vivo*, norleual suppressed the pulmonary colonization by B16-F10 melanoma in mice [119]. Thus, angiotensin-IV analogs have utility as agents in metastatic disorders via the interference with HGF–MET systems.

There are now numerous candidates for HGF-antagonistic and MET-targeted strategies (Table 3). Soluble MET-Sema and phage display-based peptides against MET may be useful as a decoy or masking agent [120,121]. Aspirin is a classic type COX2 inhibitor that reduces tumor malignancy via an inhibition of prostaglandin-dependent HGF production [122]. In addition, natural products included in foods or plants, such as diet-derived flavonoids [123] and green-tea catechins [124] exert unique activity that blocks HGF-mediated malignant behaviors. These drugs or natural products will produce or support anti-tumor outcomes.

## 8. Remarks and Perspective

It has been 15 years since NK4 was identified in 1997 as an HGF-antagonist that inhibits tumor-stroma interaction during metastasis. Stroma-rich cancers, such as gastric scirrhous carcinoma, show a great invasive activity, due to the ability of fibroblasts to secrete HGF (*i.e*., paracrine pathway). In addition, HGF induces epithelial-to-mesenchymal transition (EMT), a histological hallmark of malignant cancer, via AKT-mTOR pathways [125]. During EMT, tumor cells acquire HGF-transcriptional activity (*i.e*., autocrine mechanism). With regard to this, a mutation of *HGF* gene promoter regions (*i.e*., deletion of *HGF*-repressor region, *DATE*) leads to oncogenesis [126]. Cigarette nicotine is also responsible for HGF production in lung cancer [127]. Thus, not only tumor-secreted mediators [66], but also *HGF* mutation or environmental pollutants should be considered as a causal factor for tumorigenesis.

It is important to discuss practical approaches to anti-metastatic therapy, with a focus on NK4. HGF is an intrinsic repair factor that is essential for organ repair and protection [8,24]. Thus, tumor-targeting delivery systems of NK4 are recommended for the avoidance of systemic side effects. Implantation of NK4-overexpressing autologous cells may be available for this purpose. For example, bone marrow-derived mesenchymal stem cells (MSC), transfected with *NK4* cDNA *ex vivo*, are known to have migrated to tumor tissues, and such a local NK4 delivery led to the arrest of lung metastasis in a mouse model [128]. Similar results were obtained when *NK4* cDNA-transfected autologous macrophage were implanted in Meth-A sarcoma-bearing bearing mice [129]. Thus, MSC and macrophages can serve as a homing tool for tumor-specific NK4 delivery systems. Cationic geratin microspheres containing a NK4 plasmid DNA are also useful for tumor-targeting therapies with a slow release system [130].

The discovery of NK4 prompted researchers to search HGF-antagonists and MET-inhibitors as anti-tumor drugs. Clinical studies on anti-HGF antibody (AMG102) and anti-MET antibody (MetMab) are now ongoing in the US, and its safety and efficacy will be carefully evaluated. Additionally, diagnostic antibody against MET (*i.e*., MetSeek) may contribute to a real-time detection of metastatic cancers in nuclear imaging [131]. “Aberrant” production or activation of the HGF–MET axis is critical for the maintenance of CSC [90], drug resistance [132,133] and invasive growth [66,92–103]. Thus, we must confront the challenge to control this molecular pathway, with an appropriate, but not aberrant, regulation at will, for the establishment of invasion-free therapies.

## Figures and Tables

**Table 1:** Biological effects of HGF on intra-tumor cells.

Target cells	Effect	Involved mechanism	Reference
*Cancer cells*	Growth	β-catenin, Src, RAS activations	[32–34]
		FasL-Stat3 activation	[35]

	Dissociation	Cadherin endocytosis	[41–43]
		β-catenin activation	

	Migration ECM breakdown Anti-anoikis	cdc42-rac-PAK activation Induction of MMP Activations of PI3K-AKT Integrin-CD151 pathways	[44,45] [46–48] [49,50]

	Homing	Increased CXCR4	[51,52]
		Enhanced response to SDF1	

*Vascular cells*			

Endothelium	Mitogenesis	ERK1/2 activation	[53,54]
	Cancer-adhesion	Integrin-β4 involvement	[45,55]
	Permeability	Occludin downregulation	[56]
	
Pericytes	Migration	PI3K-AKT activations	[8]

*Immune cells*			

DC	Tolerogenic effects	TH1 << TH2 balance	[57]
T-lymphocytes	Anti-proliferation	Reduced IFN-γ	[8]

TH, Helper T-lymphocytes; IFN, Interferon; For other abbreviations see text.

**Table 2:** Representative studies to show beneficial effects of NK4 on distinct types of tumors in animal models

Tumor diseases	Animal model	Approach	Outcome	Reference
*Digestive system*				
Gastric carcinoma	TMK1 cells, ip (Mouse)	Adeno-NK4, ip	Inhibition of growth, Anti-metastasis, Anti-angiogenesis, Reduced ascites	[93]
Hepatic carcinoma	HUH7 cells, portal vein (Mouse)	Adeno-NK4, iv	Inhibition of growth, Anti-angiogenesis, Prolonged survival	[94]
Gallbladder carcinoma	GB-d1, sc (Mouse)	r-NK4, sc	Inhibition of growth, Anti-invasion	[16]
Pancreatic cancer	SUIT-2 cells, intra-pancreas (Mouse)	r-NK4, ip	Inhibition of growth, Anti-metastasis, Anti-angiogenesis, Reduced ascites, Prolonged survival	[79]
Colon carcinoma	MC-38 cells, intra-spleen (Mouse)	NK4 cDNA, bolus iv (hydrodynamics)	Inhibition of growth, Anti-metastasis, Anti-angiogenesis, Prolonged survival	[95]
*Respiratory tissue*				
Lung carcinoma	Lewis lung cancer, sc (Mouse)	r-NK4, sc	Inhibition of growth, Anti-metastasis, Anti-angiogenesis	[54]
Lung carcinoma	A549 cells, sc (Mouse)	Adeno-NK4, intra-tumor or ip	Inhibition of growth, Anti-angiogenesis	[96]
Mesothelioma	EHMES-10 cells, sc (Mouse)	Adeno-NK4, intra-tumor	Inhibition of growth, Enhanced apoptosis, Anti-angiogenesis	[97]
*Reproductive organ*				
Prostate carcinoma	PC-3 cells, sc (Mouse)	r-NK4, sc (osmotic pump)	Inhibitions of growth, Anti-angiogenesis	[98]
Ovarian carcinoma	HRA cells, ip (Mouse)	NK4 gene, Stable transfection	Anti-metastasis, Prolonged survival	[99]
*Blood system*				
Lymphoma	E.G7-OVA cells, sc (Mouse)	Adeno-NK4, intra-tumor (with DC)	Inhibition of growth, Anti-angiogenesis, Induction of CTL	[100]
Multiple myeloma	KMS11/34 cells, sc (Mouse)	Adeno-NK4, im	Inhibition of growth, Anti-angiogenesis, Enhanced apoptosis	[101]
*Others*				
Melanoma	B16F10 cells, sc (Mouse)	Adeno-NK4, iv	Inhibition of growth, Anti-metastasis, Anti-angiogenesis	[81]
Glioblastoma	U-87 MG cells, intra-brain (Mouse)	r-NK4, intra-tumor	Inhibition of growth, Anti-angiogenesis, Enhanced apoptosis	[102]
Breast carcinoma	MDAMB231 cells, sc (Mouse)	r-NK4, sc	Inhibition of growth, Anti-angiogenesis	[103]

Adeno-NK4, adenoviral vector carrying NK4 cDNA; r-NK4, recombinant NK4 protein; sc, subcutaneous; iv, intravenous; ip, intraperitoneal; im, intramuscular; DC, dendritic cells; and CTL, cytotoxic T cells.

**Table 3:** Therapeutic effects of other HGF-antagonists or MET-inhibitors on experimental tumors in animals.

Tumor diseases	Animal model	Treatment	Outcome	Reference
*Anti-HGF approaches*				
HGF knock-down	U87 glioblastoma, brain (Mouse)	HGF ribozyme, cell implant (brain)	Reduced mass size, Anti-proliferation	[104]
Uncleavable pro-HGF	MDA-MB435 breast cancer, sc (Mouse)	Pro-HGF cDNA, lentivirus vector, intra-tumor, 18 days	Anti-proliferation, Anti-angiogenesis, Enhanced apoptosis	[106]
Anti-HGF antibody	U118 glioblastoma, sc (Nude mice)	Anti-HGF IgG, sc, 2 times/week × 10	Reduced mass size	[105]
Anti-HGF antibody (AMG102) *	U87 glioblastoma, sc (Mouse)	Anti-HGF IgG, sc, 2 times/week × 5	Anti-proliferation, Enhanced caspase-3	[110]
*MET-inhibitions*				
Anti-MET antibody (MetMab) **	U87 glioblastoma, brain (Mouse)	Antibody, intra-brain, pump, 4 weeks	Anti-proliferation, Enhanced apoptosis	[111]
Anti-MET antibody (DN30)	GTL16 gastric cancer, sc (Mouse)	Antibody, sc, 2 times/week × 4	Anti-proliferation, MET shedding	[112]
Anti-pro MET antibody (LMH-80)	U87 glioblastoma, brain (Mouse)	Antibody, 3 times, sc	Anti-proliferation, Binding to pro-MET	[113]
Decorin	A431 epidermoid cancer, sc (Mouse)	5 mg/kg/48 hr, ip, 12 times	Growth arrest, β-catenin inactivation	[118]
Angiotensin-IV (Norleual)	B16F10 melanoma, iv (Mouse)	50 μg/kg/day, ip, 14 days	Anti-metastasis, Gab1 inactivation	[119]
PHA665752	NCI-H69 lung cancer, sc (Mouse)	16.5 μg/day, intra-tumor, 8 days	Anti-angiogenesis, Increased TSP-1	[115]
MK-2461 ***	GTL16 gastric cancer, sc (Mouse)	200 mg/kg/day, po, 20 days	MET Y-1349 inhibition, Growth arrest	[116]
Apigenin (Flavonoids)	MDA-MB231 breast cancer, iv (Mouse)	40 μM, iv, with cancer cells + HGF	Anti-metastasis	[123]
EGCG (Green tea)	SCC-VII/SF, sc (Mouse)	75 mg/kg/day, ip, 21 days	Anti-proliferation, Enhanced apoptosis	[124]

TSP-1: thrombospondin-1. EGCG: epigallo-catechin-3-gallate. For abbreviations see text or other tables.

*, **, *** The safety and efficacy are now being evaluated through clinical studies (phase-I/II) [117].
